# The Perspective of Using Optical Coherence Tomography in Ophthalmology: Present and Future Applications

**DOI:** 10.3390/diagnostics15040402

**Published:** 2025-02-07

**Authors:** Mario A. Vasilescu, Mioara L. Macovei

**Affiliations:** 1 Department of Ophthalmology, “Dr. Carol Davila” Central Military Emergency University Hospital, 010825 Bucharest, Romania; laura.macovei@yahoo.com; 2 Ophthalmology Department, “Carol Davila” University of Medicine and Pharmacy, 050474 Bucharest, Romania

**Keywords:** optical coherence tomography, visible-light OCT, adaptive optics, full field, wide field, polarization-sensitive, intraoperative OCT, hand-held OCT, at-home OCT

## Abstract

Optical coherence tomography (OCT) imaging plays a major role in the field of diagnosing, monitoring, and treating ophthalmological diseases. Since its introduction in the early 1990s, OCT technology has continued to advance both in the direction of acquisition quality and technique. In this manuscript, we concentrate on actual and future applications of OCT in the ophthalmology field, reviewing multiple types of OCT techniques and systems, such as visible-light OCT, adaptative optics OCT, intraoperative OCT, wide-field OCT, and more. All of them allow better monitoring of ocular diseases, earlier and broader diagnosis, and a more suitable treatment. Furthermore, overviewing all these technologies could play a pivotal role in research, leading to an advance in understanding the pathophysiology of targeted diseases. Finally, the aim of the present review was to evaluate the technical advances in OCT and their actual and potential clinical applications.

## 1. Introduction

Optical coherence tomography (OCT) is a noninvasive imaging method that, since its appearance and clinical use, has revolutionized the field of ophthalmology. OCT operates on the principle of light interference, enabling the capture of high-resolution, cross-sectional tomographic images of ocular biological tissue, such as the cornea, retina, and choroid, at the micron level, making it a near histological research tool in vivo. Consequently, since its first introduction in 1991 [1], OCT has been rapidly adopted into clinical practice, aiding clinical investigations, including indirect ophthalmoscopy in diagnosing retinal and choroidal diseases, such as age-related macular degeneration, central serous chorioretinopathy, vascular retinal disorders, and other vitreoretinal diseases.

The first OCT system used in ophthalmology was time-domain OCT (TD-OCT) [1] and, since then, OCT technology has faced impressive advances in new techniques and sophisticated systems that, ultimately, bring improvements in the resolution, scanning speed, and structural details of biological tissues through more enhanced penetration. This is why, nowadays, OCT systems are not limited only to the retina or cornea field but also to neuro-ophthalmology, research fields, and glaucoma.

Ultimately, technological advances and emerging innovations in the OCT field lead to earlier therapeutic interventions and, most importantly, the preservation of patients’ vision. Therefore, the aim of this review is to briefly assess the current literature to provide an overview of recent developments in clinical and experimental OCT imaging in ophthalmology, as well as explore potential further applications of these recent OCT imaging methods.

## 2. Materials and Methods

A structured literature search of titles in PubMed and Frontiers was performed in July 2024. The literature search followed the updated preferred reporting items for systematic reviews and scoping review (PRISMA) guidelines [2]. The keywords used for finding relevant results were “optical coherence tomography future applications”, “advances in optical coherence tomography”, “clinical applications of optical coherence tomography”, “intraoperative optical coherence tomography”, “ophthalmology”, and all relevant synonyms, abbreviations, and combinations between these terms. The references of identified articles were manually checked to locate potentially relevant studies. All studies, articles, and systematic reviews published before 2020 were excluded. The bibliographies of the selected articles or reviews were analyzed by the authors, and if relevant or complementary articles for this review were found, they were integrated into this study regardless of the year of publication. Studies were included for full-text review and qualitative analysis if they reported on potential future clinical applications and research areas of OCT and only if a recent technology or technique of the respective OCT systems was described. All studies about OCT angiography were excluded. The authors acknowledge the possibility that the search algorithm may have inadvertently excluded significant research papers.

## 3. Results

3.0 Spectral Domain OCT (SD-OCT) and functioning principle of nowadays OCT systems.

To better understand the technological advancements of OCT systems, it is necessary to briefly describe the first OCT systems and the working principle of commercially and widely available OCT systems. First of all, OCT is a light interference-based optical technique and uses light in the near-infrared (800–1300 nm). It is based on the analysis of the light back-reflected from the sample (in this case, the eye) [3].

The functional principle behind OCT imaging is light interference [4]. The light from a low-coherence source is split into two paths by a coupler directing it along two different arms of an interferometer [4]. One arm is the reference arm, while the other arm is the sample arm [4]. In the reference arm, the light is backscattered by a reference mirror, while in the sample arm, the light is backscattered by the sample (the eye). The returning light from both arms recombines at the coupler and generates an interference pattern, which is recorded by the detector [4]. The OCT signal recorded by the detector during a complete travel of the reference mirror is called a depth scan or an A-scan. An OCT image, otherwise called a B-scan, is formed by a set of consecutive A-scans [4].

Time-domain OCT (TD-OCT) was the first OCT technology developed, and it uses a low-coherent light source and a scanning reference delay [4]. The reference mirror moves, allowing the reference light to travel a known path length and undergo a measurable variable time delay [4]. Its disadvantages were very slow imaging speed and poor image quality, all of them because it required the acquisition of a depth scan for every location [3].

The introduction of the Fourier domain OCT (FD-OCT) addressed these limitations. Perhaps the greatest difference between TD-OCT and FD-OCT is that the reference arm in an FD-OCT system has a static mirror instead of a moving one, as in TD-OCT [4]. The existence of this static mirror eliminates the limitations imposed by the inertia of a moving mirror, allowing higher acquisition speeds [4]. FD-OCT has evolved into two primary technologies: spectral domain OCT (SD-OCT) and swept-source OCT (SS-OCT).

SD-OCT uses a spectrometer. This device incorporates a diffractive element that separates the different wavelength contributions into a line image, which is then recorded by a high-speed line scan camera. Each camera read-out constitutes a spectral interferogram with a superposition of fringe patterns [3]. Typically, a superluminescent diode is chosen as a broadband light source [3].

Two years after the development of SD-OCT, the broadband light source was replaced by an optical source that rapidly sweeps a narrow line width over a broad range of wavelengths, and this is called swept-source OCT (SS-OCT) [3]. Additionally, SS-OCT has a point detector, not a line or area detector. Point detection is one advantage that SS-OCT has over SD-OCT because of its higher signal-to-noise ratio when compared to area or line detectors [4]. In contrast to TD-OCT, the interferogram contains information for all depth layers of the sample simultaneously. Fourier transformation is required to extract their individual contribution as a function of their depth position [3].

A detailed description of the technology behind SD-OCT is beyond the scope of this paper, but it is certain that the principles outlined above have driven the development of spectral OCT techniques. These advancements allow a dramatic increase in the signal-to-noise ratio (SNR) and imaging speed [5,6,7].

### 3.1. Visible-Light OCT (Vis-OCT)

#### 3.1.1. Technological Advances

The main characteristic of Vis-OCT is that it utilizes light (500–600 nm) for OCT illumination rather than near-infrared (NIR) light (800–1300 nm), and its development was primarily motivated by two considerations: (1) with comparable bandwidth, shorter illumination wavelengths improve axial resolution (<2 μm) and (2) vis-OCT can retrieve tissue scattering and absorption contrasts within the visible spectral range [8].

#### 3.1.2. Clinical Application

Yi et al. [9] reported the first vis-OCT system for human retinal imaging. When comparing vis-OCT and NIR-OCT, both modalities provided similar representations of retinal anatomy in B-scans. However, vis-OCT demonstrated significantly enhanced contrast of the retinal nerve fiber layer (RNFL) and the layers in the outer retina, including the boundary between the photoreceptor inner and outer segments (IS/OS), the OS of the photoreceptors, the RPE layers, and Bruch’s membrane.

The inner retinal layers in OCT correspond reasonably well with histological sections [10]. In contrast, interpreting the outer retinal hyperreflective bands presents challenges. These difficulties arise from the complex and intimate relationship between the retinal pigment epithelium (RPE) and photoreceptors [11], the proximity of the RPE to Bruch’s membrane (BM) [12], and the fine layers of stacked photoreceptors [13] and RPE organelles [14]. This is where vis-OCT proves advantageous.

Recent studies have shown that vis-OCT can especially delineate two structural components of the retina: Bruch’s membrane [15,16,17] and the inner plexiform layer (IPL) sublayers [17,18]. High-resolution visualization of the outer retinal band 4, especially the Bruch’s membrane, would be helpful for monitoring macular degeneration diseases. This capability also holds promise for the development of new classification systems for such conditions. Clinically, vis-OCT could play a pivotal role in determining the optimal timing for initiating treatment and monitoring responses to different therapeutic approaches, particularly when we refer to age-related macular degeneration. Additionally, the visualization of the inner plexiform layer, which reflects scattering information from dendritic connections, has the potential to serve as a biomarker for glaucoma progression [19].

Another potential clinical application of VIS-OCT was remarked by Song W. et al. [20]. They quantified the reflectivity of the peripapillary retinal nerve fiber layer (pRNFL). The pRNFL of all eyes—normal, suspect/preperimetric glaucoma (GS/PPG), and perimetric glaucoma (PG)—were scanned by a custom-designed dual-channel device that simultaneously acquired VIS-OCT and NIR- OCT. The results showed that VIS-OCT pRNFL reflectivity more effectively distinguished GS/PPG from normal eyes compared to NIR- OCT. This finding highlights the clinical relevance of vis-OCT as a tool for the early detection of glaucoma.

In addition, vis-OCT can evaluate quantitatively the oxygen saturation (sO_2_) in retinal circulation [8]. Yi J. et al. [21] reported that vis-OCT, as a standalone imaging mapping modality, could quantify a full set of the metabolic parameters of retinal circulation, including total retinal oxygen delivery, oxygen extraction fraction, and metabolic rate of oxygen. These parameters could be utilized clinically to grade diseases affecting retinal circulation, such as diabetic retinopathy, retinopathy of prematurity (RoP), and retinal occlusive diseases.

Another clinical use of vis-OCT ability to measure retinal oximetry was suggested by Wang J. et al. [22]. They concluded that GS/PPG and PG subjects exhibited significantly higher macular venous oxygen saturation, a reduced difference between arteriolar and venular oxygen saturation, and lower oxygen extraction, indicating less oxygen consumption of these eyes. These findings suggest that retinal oximetry obtained via vis-OCT could serve as potential metrics for the early diagnosis of glaucoma.

In addition to these clinical applications, vis-OCT has potential utility in fundus autofluorescence (FAF) imaging [8]. Since vis-OCT can integrate with FAF imaging without requiring an additional light source, it enables the quantifications of true RPE autofluorescence (AF) intensity [23]. This is achieved by calculating the ratio of the vis-OCT signal intensity and AF signal intensity at the RPE [23].

Furthermore, due to the shorter illumination wavelength of vis-OCT, it is possible to retrieve the distribution of rhodopsin [24]. Rhodopsin is a functional biomarker of rod photoreceptors. Such evaluation has significant clinical implications. For instance, monitoring rhodopsin expression in photoreceptors may aid in the management of hereditary retinal degeneration (including retinitis pigmentosa) or AMD [24].

### 3.2. Adaptative Optics OCT (AO-OCT)

#### 3.2.1. Technological Advances

The use of adaptative optics in ophthalmology was inspired by astronomical observations [25]. In astronomy, AO is employed in telescopes to obtain diffraction-limited imaging from the ground by compensating for the dynamic wavefront distortion caused by atmospheric turbulence. Like the atmosphere, the human eye is optically imperfect. It consists of the cornea, the anterior chamber, the lens, the vitreous body, and the retina. All these media are passed through by light, causing chromatic and monochromatic aberrations. Adaptative optics specifically address monochromatic aberrations, which are categorized into low-order and high-order aberrations. The mechanism which limits this phenomenon is the adjusting of pupil size, so in a daily life, people can see objects clearly. However, in conventional imaging systems without AO, which aim to capture details of the retina, such natural adjustments are not possible.

In retinal imaging, the diffraction-limited optical resolution is governed by the numerical aperture (NA) of the human eye. The NA is limited to 0,24 due to the focal length of the eye and the maximum pupil diameter (approximately 8 mm) [3]. According to the diffraction limit formula, r = 0.61 *λ*/*NA* (*r* is the resolution, *λ* is the imaging wavelength, and *NA* is the numerical aperture) [26], a theoretical resolution of about 2 μm can be achieved [3,26]. However, in practice, optical aberrations of the human eye increase with the pupil diameter. Therefore, commercial OCT systems typically offer a lateral resolution of about 9 μm [3] to 15 μm [26] by sending into the eye a beam of 2 mm in diameter [3].

In essence, it could be said that AO is for the fundus imaging techniques, what the adjusting of the pupil size is for the eye. It actively compensates for optical aberrations through an adaptive optical component like, e.g., a deformable mirror [3].

The primary advantage of AO-OCT is that the 2 μm axial resolution is actually a cellular resolution. Consequently, this leads to the ability to visualize in vivo the cellular-sized structures within the human retina, such as nerve fibers, photoreceptors, RPE, or capillaries. This feature is particularly relevant for research purposes.

AO-OCT is not widely used in clinical ophthalmology due to several limitations, including motion artifacts, the need for constant fixation, poor mydriasis, and the presence of any media opacities that significantly degrade the image quality [27]. Despite these challenges, Mozaffari S. et al. [28] addressed some of these limitations and described a system of eye tracking that could remove eye motion artifacts.

#### 3.2.2. Clinical Application

The visualization of photoreceptors allows for the monitoring and diagnosing of diseases that affect the retinal layer. For instance, it can be utilized in inherited retinal degeneration, including but not limited to retinitis pigmentosa, cone-rod dystrophy, Stargardt’s macular dystrophy, choroideremia, and Usher’s syndrome and achromatopsia [29]. Additionally, it holds significant value in AMD. Several studies have demonstrated alterations in photoreceptor density and reflectivity at retinal sites overlaying drusen [30,31] and subretinal drusenoid deposits [32,33].

AO-OCT further enables visualization of the ganglion cell bodies [34]. Thanks to the 3D micrometric resolution of AO-OCT, a full 3D organization of the retinal ganglion cells (RGC) mosaic was revealed. Quantifying the RGC morphology offers a promising perspective in studying and diagnosing glaucoma [35].

Moreover, initial changes in inner retinal thickness in glaucoma may be followed by outer retinal changes. These include changes to the integrity and density of cone photoreceptors. Photoreceptors’ outer segments are observed to be shorter and exhibit greater variability in retinal areas associated with visual field loss compared to unaffected or less affected areas [36,37].

In the context of glaucoma, AO-OCT also allows direct visualization of the lamina cribrosa. Its high resolution allows a microscopic point of view of its network of collagenous beams at different axial depths [38,39]. Elevations in intraocular pressure contribute to the mechanical reshaping of the lamina cribrosa’s microarchitecture, which AO-OCT can effectively capture.

Regarding retinal vasculature, clinical imaging of choriocapillaris is hard to obtain due to its dense capillary network and the overlaying melanin-rich RPE. Both block light from reaching the target. AO-OCT angiography allowed the in vivo imaging of the choriocapillaris structure and perfusion in the human retina [40]. These imaging approaches could track choriocapillaris changes, such as the disruptions found in AMD.

Ultimately and most recently, AO-OCT is applied to monitor responses to emerging treatments, including gene and small-molecule therapies [41], stem cell transplantation [42], and optogenetic therapies [43]. Its cellular-level resolution facilitates detailed assessment, further solidifying its role in advancing ophthalmic research and clinical practice.

### 3.3. Polarization-Sensitive OCT

#### 3.3.1. Technological Advances

In the case of OCT, light is emitted to visualize tissues. To enhance tissue contrast, various physical properties of light are exploited, such as the wavelength or the intensity. Another important property of light is polarization.

Within the eye, all biological tissues that light passes through (including the light emitted by conventional TD- and SD-OCT) contain varying amounts of collagen. The polarization state of light is altered along the axial depth when passing through these collagenous tissues [44]. So, an additional contrast could be provided by measuring and evaluating the change in the polarization state of the backscattered probe light [3].

PS-OCT can analyze the birefringence, providing quantitative information related to the polarization-dependent changes [45], and in 1992, the functions of PS-OCT were first demonstrated [46].

#### 3.3.2. Clinical Application

Given PS-OCT’s ability to increase the contrast of fibrous tissues, it is suitable for imaging diseases, which imply collagen-reach structures (for example, the cornea and sclera), fibrosis growth (for example, AMD), or retinal structures, such as RPE.

Clinically, PS-OCT is worthy of being applied for the investigation of glaucoma, including measuring the internal space of filtering blebs following trabeculectomy [47,48,49,50,51] or for the noninvasive visualization of the trabecular meshwork (TM) [52]. It is noteworthy that in addition to these structures of interest for glaucoma study, Schlemm’s canal observation through PS-OCT is not suitable because it is a hollow space (a tubular canal) rather than a solid collagenous tissue [44].

PS-OCT was found useful for the assessment of keratoconus [53,54,55]. The better ability of PS-OCT for imaging the cornea could offer, in the future, the assessment of cornea degeneration, keratitis, or corneal leucoma development.

RPE is a melanin-containing structure [56] and depolarizes. Therefore, PS-OCT imaging offers a better contrast of RPE, making its visualization clearer. RPE is significantly involved in the pathogenesis of neovascular AMD, and the identification of disease-specific patterns in the choroidal neovascular membrane is significant regarding the status and progress of RPE, potentially contributing to a better pathophysiological understanding of neovascular AMD [57]. Similarly, PS-OCT can segment the geographic atrophy seen in the dry form of AMD [58]. Moreover, the retinal fibrosis growth in the setting of neovascular AMD can be tracked using PS-OCT [59].

In addition, PS-OCT has an adaptation, namely, a polarization-sensitive quantum (PS-QOCT), which provides dispersion cancellation and identifies the refractive index of a media [60]. Thus, PS-QOCT provides both an improved resolution and improved contrast of collagenous fibers [61,62].

### 3.4. Full-Field OCT (FF-OCT) and Dynamic FFOCT

#### 3.4.1. Technological Advances

FF-OCT enables the acquisition of histology-like images of a sample noninvasively, offering controllable transverse and axial resolution ranging from 10 μm to as fine as 1 μm, with an imaging depth of 1 mm or more, depending on the scattering characteristics of the samples [63]. This capability is achieved through the combination of enhanced lateral resolution and axial resolution. The lateral resolution is improved by employing lenses with a high NA [63], while the axial resolution benefits from the use of a broadband light source [63]. With these technical improvements, FF-OCT collects interferograms of different phases at the focusing depth of the sample by the planar detector [63].

FF-OCT provides isotropic micrometric resolution and has the ability to produce a histology-like image from a sample within a few milliseconds without physical slicing, making this investigation a form of “optical biopsy” [63]. This capability enables the identification of the features from the microstructural to cellular level, making it valuable across various medical fields, including ophthalmology, oncology, and dermatology. In addition to ophthalmology, the reported applications include the discrimination of the benign and malignant tissues of skin cancer [64,65], breast invasive ductal cancer [66,67], and head and neck cancer [68], as well as the optical biopsy of intestinal tissues [69,70].

#### 3.4.2. Clinical Application

In ophthalmology, FF-OCT enables the visualization of retinal structures, such as the nerve fiber layer, ganglion cell layer (GCL), inner plexiform layer (INL), outer plexiform layer (OPL), and outer nuclear layer (ONL). Due to its subcellular resolution, FF-OCT can also detect the cellular characteristics of each layer within the retina, such as the details of ganglion cell axons and cell bodies [63,71].

The FF-OCT presents some limitations in acquiring images of in vivo human retinal structures due to the presence of continuous involuntary head and eye axial motion and a relatively small field of view (FOV). Viacheslav M. et al. demonstrated a potential use of FF-OCT in the ophthalmology field [72]. Also, Mecê P. et al. developed a system that optically compensates for axial motion in real-time for time-domain FF-OCT [73].

Mazlin et al. [74] allowed visualization of the FOV of the corneal sub-basal nerve and endothelial layers. Many more nerves and cells could be counted. This could potentially lead to a more precise quantitative diagnosis of corneal conditions [75].

A derivative of FF-OCT has been described as providing the visualization of dynamic structures at the microscopic level. In the dynamic FF-OCT (D-FFOCT), backscattered light from subcellular structures in motion can be measured in a time-dependent fashion, which potentiates live or time-lapse imaging [76]. D-FFOCT can noninvasively capture mitochondrial dynamics and pigment distribution in RPE cells in real time [77]. Groux et al. [77] showed that D-FFOCT allows the imaging and study of degeneration in RPE cell cultures. Thus, it may play a pivotal role in understanding and monitoring degenerative retinal diseases (such as AMD). Furthermore, it may become a tool for following such diseases in a microscopic manner in vivo. Moreover, D-FFOCT could be applied to study optic nerve diseases, as mitochondrial dynamics, activity, and fragmentation play a key role in these conditions [77,78].

Another recent application of D-FFOCT is described by Scholler et al. [79], who introduced a novel method of the label-free imaging of retinal organoids using D-FFOCT and used it to monitor the temporal development of retinal organoids with a temporal resolution of 20 ms.

### 3.5. Intraoperative OCT (iOCT)

#### 3.5.1. Technological Advances

The first clinical application of iOCT was reported in 2005 using a handheld OCT device during anterior segment surgery [80]. Since then, technology has evolved and made systems more and more mobile and compact. However, the implementation of iOCT in clinical settings faces several challenges, such as a sterile environment, delayed surgical workflow, significant investment, and, most importantly, a still limited understanding of the benefits, referring to the balance between surgeons’ decisions and consequently, the postsurgical evolution of the patients [81].

To address this, Ehlers et al. conducted two studies in order to investigate the impact of iOCT on clinical decision-making: prospective and perioperative ophthalmic imaging with optical coherence tomography (PIONEER) [81] and Determination of the Feasibility of Intraoperative Spectral Domain Microscope Combined/Integrated OCT Visualization During En Face Retinal and Ophthalmic Surgery (DISCOVER) [82]. In both studies, the iOCT images led to an altered surgical decision in anterior and posterior segment surgery. For example, the completeness of retinal membrane peeling and adherence of posterior lamellar keratoplasty grafts are some of the surgical information provided by iOCT, which can alter surgical decision-making [81].

Regarding devices incorporating an iOCT system, technological advances have made possible three types of devices, which are currently used in practice: handheld, microscope-integrated, and instrument/probe-integrated [83].

Currently, iOCT is widely used in various types of surgeries: vitreoretinal surgery, corneal surgery, cataract surgery, glaucoma surgery, corneal crosslinking, refractive surgery, and pediatric ophthalmic surgery [83].

#### 3.5.2. Clinical Application

##### Posterior Segment Surgery

In vitreoretinal surgery, the current applications of iOCT are routine surgeries, such as retinal membrane peeling, macular hole surgery, and retinal detachment surgery [83], especially in complex cases [84]. However, iOCT aids in managing challenging vitreoretinal interventions that entail a high risk of misplacement, incorrect removal of tissue, or scar tissue formation, which suggests the potential future directions of its clinical use.

Beyond enhancing the visualization of anatomical structures during surgery and guiding through the surgical procedure, iOCT provides a predictability value regarding postoperative outcomes. For example, during the peeling process of the epiretinal membrane, retinal stress and thickening are induced. The amount of retinal thickening immediately following peeling was measured with iOCT in a study [85]. They found a correlation between retinal thickening and postoperative development of a dissociated optic nerve fiber layer (DONFL) [85].

iOCT also plays a role in the submacular space. Kashani A.H. et al. [86] demonstrated that iOCT could confirm the successful implantation of a biosynthetic RPE monolayer for treating geographic atrophy. Also, iOCT visualizes and confirms the position of the cannula in the subretinal space for direct drug delivery [87,88]. Even more, in cases of maximum precision, such as delivering drugs directly between photoreceptors and the retinal pigment epithelium, iOCT is a mandatory tool [89]. Given the dimension of the subretinal space and the surgical precision of its approach, iOCT can be a tool used in robotic-assisted subretinal delivery [90]. In addition to these kinds of procedures, iOCT can be used for retinal prosthesis implantation and chorioretinal biopsies [91].

In a study, Asahina Y. et al. [92] analyzed 20 eyes with a clinical diagnosis of diabetic retinopathy and refractory cystoid macular edema (CME) treated with iOCT-guided cystotomy. The results indicated that iOCT-guided cystotomy could be another treatment option for refractory CME in diabetic eyes. Also, iOCT proves useful in retinal biopsies [93] and subretinal and submacular injections [88].

Furthermore, iOCT can assess the effect of instrumentation on tissues during vitreoretinal surgeries [94]. Since surgeons lack tactile feedback during such procedures, iOCT B-scans provide visual cues on how tissues react to different maneuvers using various instruments.

Furthermore, iOCT allows the assessment of fovea status in cases of vitreous opacities, such as vitreous hemorrhage, which obstruct preoperatively fovea visualization due to the shadowing effect [94].

##### Anterior Segment Surgery

In anterior segment surgery, iOCT is frequently used in different types of keratoplasties: deep lamellar anterior keratoplasty (DALK), Descemet stripping endothelial keratoplasty (DSEK), and Descemet membrane endothelial keratoplasty (DMEK) [95]. During these keratoplasties, through iOCT, the surgeon can directly assess the depth of the dissection planes if necessary [95]. The use of iOCT during DMEK aids the surgeon in orientating, positioning, or unfolding the graft [96,97]. Patel et al. [96] reported that iOCT can improve surgical efficiency by visualizing these maneuvers, leading to a decrease in the duration of these maneuvers and graft handling and minimizing endothelial cell loss.

For corneal trauma and other corneal surgeries, iOCT demonstrates its helpfulness. Eguchi H. et al. [98] reported that iOCT can facilitate securing corneal sutures by demonstrating the needle depth during suture procedures; can assess the depth of foreign bodies in the cornea, thereby facilitating effective removal and reducing the risk of complications; and can precisely estimate the depth of corneal opacities in cases of chemical or thermal burns. Also, in cases of surgical treatment for Salzmann nodular degeneration, iOCT can visualize the cleavage plane between the nodule and underlying corneal tissue in image-guided lamellar keratectomy [82]. For the treatment of advanced keratoconus, iOCT can be used in a new procedure, namely, Bowman layer transplantation, even when the air–endothelial interface is obscured [99].

##### Glaucoma Surgery

Regarding glaucoma surgeries, Muijzer et al. [83] concluded that, overall, the currently available iOCT devices do not show an important impact on targeted tissue (such as the trabecular meshwork or sclera) visualization and, therefore, a clinical benefit. The research algorithm used in this paper also led to their conclusion.

##### Cataract Surgery

Regarding cataract surgery, iOCT has demonstrated its utility in assessing the most important aspects of this type of surgery: surgical safety and the refractive outcome. Regarding safety and referring especially to young surgeons, iOCT provides direct visualization of the groove depth and the construction of self-sealing corneal incisions [100,101]. IOCT can confirm the placement of the intra-ocular lens (IOL) in the capsule bag [101] and can detect capsular defects [102].

Additionally, even experienced cataract surgeons could benefit from the use of iOCT. For example, in cases of posterior polar cataracts, iOCT detects pre-existing posterior capsular dehiscence during cataract surgery, thereby preventing lens drop [102]. Also, during IOL scleral fixation, iOCT visualizes the sclera flap preparations and can assess the preparation depth, which results in enhanced stability of the fixated IOL [103].

Regarding posterior pole cataracts, Sarkar S. et al. [104] investigated the efficacy of preoperative anterior segment OCT to predict intraoperative posterior capsular rupture (PCR). They found that the sensitivity and specificity of anterior segment OCT for detecting dehiscence were 94.4% and 87.5%, respectively. In other words, anterior segment OCT can be used in a diagnostic manner for this type of complication in this special type of cataract.

Regarding the refractive outcome of cataract surgery, the images from iOCT and the associated data regarding the IOL position can optimize IOL calculations and future IOL designs. The meridian lens position has a great influence over the anterior chamber depth (ACD) [105]. Thereby, a study found that integrating iOCT data into current IOL power calculation formulas can improve refractive outcomes [106]. Similarly, the intraoperative ACD measured using OCT was a significantly better predictor for postoperative-manifesting refractive outcomes [106,107,108]. Furthermore, Hirnschall et al. showed 7.1% of cases would have resulted in a different IOL power if preoperative and intraoperative ACD measurements had been combined [106]. During IOL scleral fixation, iOCT can also be helpful in the adjustment of the IOL tilt, thus reducing potential residual cylinder diopters [109].

### 3.6. At-Home OCT

There are some retinal diseases (such as neovascular AMD or retinopathy of prematurity (ROP)) that require periodical monitoring via clinic visits and OCT imaging to survey disease evolution and ensure proper management. These visits can often be burdensome, especially for the elderly population [110]. Several studies have shown that the use of at-home OCT was feasible among patients and was clinically gradable and, therefore, successful in monitoring macular diseases and response to treatments [110,111,112,113].

Liu Y. et al. [110,111] studied the results of regular self-imaging through a novel spectral domain OCT system (Notal Vision Home OCT). They gathered several parameters, such as retinal fluid volume and fluid load over time, among patients suffering from neovascular AMD and compared the images taken by the patients with the images acquired by the medical personnel. They concluded that the novel system meets the requirements for self-controlled imaging, and it can be used in the future for illustrating the acute changes in neovascular AMD and anti-VEGF therapy responses.

Furthermore, Nahen K. et al. [111] concluded that image analysis based on artificial intelligence can potentially support clinicians in the assessment and utilization of a large amount of data generated by daily home OCT imaging. Also, Burchard et al. [112,113] obtained similar results with a different at-home OCT system: a low-cost full-field based OCT with realistic production costs below USD 1000. Moreover, they scanned diabetic macular edema and retinal vein occlusion in addition to neovascular AMD.

### 3.7. Hand-Held OCT (HH-OCT)

This type of OCT technology has been developed to address the main practical limitation of commercially available OCTs, namely, limited portability. Therefore, they serve as an efficient and diagnostic point-of-care imaging tool [114].

Thanks to its portability, HH-OCT can address limitations for patients who cannot be scanned by commercial OCT systems: bedridden patients [115] and infants or young populations [116,117,118]. However, given the fact that this sort of system does not present a stiff support (such as a table), Wang K.L. et al. [119] remarked on several challenges for a handheld OCT system, including operator variability, hand movement, and manual alignment.

Nicholson R. et al. [117] demonstrated that OCT imaging of children with Down syndrome in an outpatient setting is feasible, revealing a high incidence of fovea hypoplasia in this group, associated with thickening of the ganglion cell and inner nuclear layers at the fovea.

Kubsad D. et al. [120] used HH-OCT to evaluate several ROP severity vitreoretinal biomarkers, such as punctate hyperreflective vitreous opacities and vitreous bands, foveal development biomarkers, choroidal features, and dome-shaped macula. These biomarkers showed subclinical structural findings, and they were associated with the development and progression of ROP. These findings make HH-OCT a useful tool for guiding screening and treatment strategies.

Also, Mangladesh S. et al. [121] examined infants at the bedside in an intensive care nursery and compared the stress level of the infants induced by the HH-OCT imaging investigation and binocular indirect ophthalmoscopy (BIO) examination for ROP. They showed that investigational HH-OCT imaging is less stressful for infants than BIO examination.

### 3.8. Wide-Field and Ultrawide-Field OCT (WF-OCT and UWF-OCT)

#### 3.8.1. Technological Advances

Standard OCT systems are constrained to a relatively narrow field of view (FOV) of around 30 × 30 degrees [122]. This is why patients are asked to move their eyes to scan the peripheral retina and widen the FOV. To address this limitation in imaging capability, wide-field OCT technology with an FOV of around 40–55 degrees and ultrawide-field OCT with a FOV of up to 200 degrees were developed [123,124].

In 2019, the International Widefield Imaging Study Group presented its definitions of wide-field imaging. A wide field was classified as an FOV of 60–100 degrees. This means the mid-periphery of the retina up to the posterior edge of the vortex vein ampulla. From the anterior edge of the vertex vein ampulla and beyond to the ora serrata was defined as an ultrawide field, meaning an FOV of 110–220 [125].

#### 3.8.2. Clinical Application

This type of OCT can easily scan the peripheral retina and allows for capturing peripheral retinal diseases, such as retinal tears, retinal holes, retinal tuft, retinoschisis, choroidal lesions, vitreous detachments, or lattice degeneration [126].

UWF-OCT could be used to assess posterior vitreous detachments in high-myopia eyes. Takahashi H. et al. [123] demonstrated that vitreous traction spanning a wide distance may lead to myopic traction maculopathy. Also, in high-myopia eyes, only WF-OCT could detect the posterior staphyloma’s presence, depth, and perimeter. Specifically, the projection image could better see the edge of the staphyloma as a dark ring [122].

WF-OCT was able to scan peripheral choroidal lesions with superior quality than standard OCT, providing information on their size, shape, distance from the optic nerve and macula, and impact on the choriocapillaris and choroid. Furthermore, it revealed no vascularity in benign nevi and tubercular granuloma but dense vascularity in melanoma and choroidal hemangioma [122].

Ripa M. et al. [122] acquired WFT-OCT scans in 311 eyes. In this group, 39% had peripheral lesions, and 31% were not detectable using slit lamp biomicroscopy, WF retinography, and standard macular OCT. This reveals the potential complementary role of UWF-OCT when examining the peripheral retina. Also, in their study [122], WF and UWF-OCT revealed the location and extent of the retinal detachment, the retinal tear locations, and preretinal membranes in all eyes with retinal detachment.

Furthermore, UWF-OCT could be used in cases with various densities of vitreous hemorrhages. Retinography, standard macular OCT, and slit lamp biomicroscopy cannot thoroughly examine the fundus. Blood’s cellularity is perceived as a collection of numerous aggregate dots. The vitreous cellularity in five uveitic eyes was remarkably similar to that originating from blood. Moreover, the cellularity could be detected neither with retinography nor standard macular OCT [122].

WF-OCT imaging was incorporated successfully into a hand-held device, as presented by Nyugen et al. [127]. They obtained high-resolution scanning images in pediatric populations. Their FOV was 105 degrees. Therefore, they obtained diagnoses and peripheral lesions, such as ROP, tractional retinal detachments secondary to ROP, rhegmatogenous retinal detachment, chronic exudative retinopathy, persistent fetal vasculature, incontinenta pigmenti with peripheral avascular retina, x-linked retinoschisis, and chorioretinal scarring with retinal traction secondary to non-accidental trauma and coats disease.

Regarding future applications in ophthalmology, WF-OCT and UWF-OCT could undergo the application of artificial intelligence, which is playing an increasingly important role in optimizing the delivery of research and diagnoses in ophthalmology [128].

WF-OCT and UWF-OCT are not without challenges. Increasing optical aberrations as the FOV increases, pupillary and ciliary shadowing-related artifacts, increased peripheral retinal curvature and inter-individual variation in retinal curvature, and the need for a very high A-scan rate (>1 million A-scans per second) represent multiple variables that need to be addressed to obtain analyzable dense WF-OCT scans [60].

To summarize, WF-OCT and UWF-OCT provide high-quality, noninvasive, in vivo tomographic details of chorioretinal layers, aiding clinicians in diagnosing and managing peripheral chorioretinal disorders.

### 3.9. Summarized Discussions

Table 1 summarizes the main technical features and, subsequently, the main benefits of tissue scanning and visualization. Table 2 shows the areas where the discussed OCT technologies have clinical applications.

## 4. Discussions

After evaluating the clinical use of these technological advancements in OCT systems, it is essential to briefly discuss their actual and potential uses. These advancements clearly address several pressing needs in ophthalmology. All the OCT systems discussed above emerged as responses to the limitations of conventional SD-OCT, which remains the most widely available OCT system in clinical ophthalmology today.

To better understand their actual roles in ophthalmology, we categorized these systems based on the specific needs they address. The need for enhanced contrast is met by the vis-OCT and PS-OCT, and AO-OCT and FF-OCT address the need for a higher resolution. Meanwhile, systems like iOCT, at-home OCT, HH-OCT, WF-OCT, and UWF-OCT integrate conventional SD-OCT into various devices, overcoming limitations such as portability, visualization of the peripheral retina, scanning bedridden patients, and regular monitoring of chronic diseases.

It is also important to distinguish between their clinical applications and their role in research. For instance, AO-OCT and FF-OCT are particularly suited for research purposes due to their high resolution. In contrast, iOCT, at-home OCT, WF-OCT, UWF-OCT, and HH-OCT are primarily designed for clinical use. Their advancements lie in the integration of SD-OCT into specialized devices rather than innovations within the OCT system itself. Finally, vis-OCT and PS-OCT serve as versatile imaging tools with significant potential for both clinical and research applications.

Regarding the aim of this paper—namely, examining the current and potential application of these OCT advances—several factors must be considered.

First, there is the distinction between strictly clinical ophthalmology and surgical ophthalmology. iOCT has already proven itself as a well-known useful tool in various ophthalmic surgeries. We have discussed above its actual application. Its future applications will likely focus on refining the surgical outcomes, such as improving refractive results in anterior segment surgeries or enhancing visual recovery following manipulations of posterior structures during posterior segment procedures.

Second, regarding the clinical use of these OCT technologies, it is necessary to recognize that they are not yet widely available or accessible in ophthalmology clinics. The reason for that is, for instance, that vis-OCT, PS-OCT, and AO-OCT provide detailed imaging capabilities that could pave the way for new disease classifications or influence the timing of treatments for conditions like retinal diseases or glaucoma. These developments raise broader considerations beyond clinical applications, such as evaluating the clinical benefit and cost-effectiveness of these systems. These complexities further blur the boundaries between their current clinical utility and their future potential.

## 5. Conclusions

OCT is a powerful tool for diagnosing and monitoring diverse diseases in ophthalmology. Its technology progresses continuously. New advances reflect the need to overcome some limitations in understanding and, finally, treating ophthalmological pathologies. Nowadays, OCT offers a large spectrum of imaging support starting from greater resolution, which can achieve histological resolution and result in intraoperative support and portability in patients’ homes. The diversity of OCT’s innovations also covers the diversity of ocular diseases, being complementary for diagnostics. In addition, widening this spectrum of innovations could be enriching for research and, consequently, early treatment. Furthermore, artificial intelligence could play a major role in ophthalmology through OCT imaging. As technology continues to develop, OCT represents a promising imaging method to assist more and more the medical act.

## Figures and Tables

**Table 1:** The main technical advances and the main benefits of each OCT technology.

Advances in OCT	Main Technical Characteristic	Main Benefit	Reference
Visible-light OCT	It utilizes light (500–600 nm) for OCT illumination	Improved axial imaging resolution (<2 μm) Quantitative evaluation of oxygen saturation in retinal circulation	[8]
Adaptative optics OCT	It limits the wavefront aberrations	Cellular resolution imaging	[26]
Polarization-sensitive OCT	It analyzes the birefringence, providing quantitative information related to the polarization-dependent changes	Suitable for imaging diseases that imply collagen-reach structures	[45]
Full-field and dynamic OCT	It is based on the principle of low-coherent interference	It provides histology-like images of a sample noninvasively with a controllable transverse and axial resolution (10 μm or even 1 μm)	[63]
Intraoperative OCT	Portable OCT system	OCT imaging during surgeries	[83]
At-home OCT	Portable OCT system	Self-imaging by patients	[110]
Hand-held OCT	Portable OCT system	Suitable for bedridden patients and pediatric patients	[115,116]
Wide-field and ultrawide-field OCT	Increasing field of view up to 200 degrees	Scans peripheral retina	[123,124]

**Table 2:** The field of clinical application of each OCT technology.

OCT Technologies	Fields of Clinical Application
Visible-light OCT	Glaucoma [19,20,22]; Vascular retinopathies [8,21]; Retinal degeneration [24].
Adaptive optics OCT	Retinal degeneration [29,30,31,32,33]; Glaucoma [35,36,37,38,39]; Monitoring gene and small-molecule therapies [41], stem-cell transplantation [42], and optogenetic therapies [43].
Polarization-sensitive OCT	Glaucoma [47,48,49,50,51,52]; Keratoconus [53,54,55]; Neovascular AMD [57,59] and geographic AMD [58].
Full-field and dynamic full-field OCT	Corneal diseases [75]; Retinal degeneration [77].
Intraoperative OCT	Posterior segment: retinal membrane peeling, macular hole surgery, retinal detachment surgery [83,84], submacular surgeries and interventions [86,87,88,89,90,91], and cystotomy [92]; Anterior segment: DALK, DMEK, DSEK [95,96,97], other corneal surgeries [82,98], and keratoconus (Bowman layer transplantation) [99]; Cataract surgery: surgical safety [100,101,102,103,104] and refractive outcome [106,107,108,109].
At-home OCT	Periodical monitoring of retinal diseases and the response to treatments [110,111,112,113].
Hand-held OCT	Scanning bedridden patients [115], infants and young population [116,117,118]; Evaluation of ROP [120].
Wide-field and ultrawide-field OCT	Examining peripheral retina [126]; Monitoring high-myopia eyes [122,123]; Examining vitreous cellularity [122].
